# Clear speech promotes speaking rate normalization^a)^

**DOI:** 10.1121/10.0019499

**Published:** 2023-05-01

**Authors:** Lilah Kahloon, Anya E. Shorey, Caleb J. King, Christian E. Stilp

**Affiliations:** Department of Psychological and Brain Sciences, University of Louisville, Louisville, Kentucky 40292, USA

## Abstract

When speaking in noisy conditions or to a hearing-impaired listener, talkers often use clear speech, which is typically slower than conversational speech. In other research, changes in speaking rate affect speech perception through speaking rate normalization: Slower context sounds encourage perception of subsequent sounds as faster, and vice versa. Here, on each trial, listeners heard a context sentence before the target word (which varied from “deer” to “tier”). Clear and slowed conversational context sentences elicited more “deer” responses than conversational sentences, consistent with rate normalization. Changing speaking styles aids speech intelligibility but might also produce other outcomes that alter sound/word recognition.

## 1. Introduction

Picheny et al. (1985) conducted a seminal study on the effects of different speaking styles on speech intelligibility for hearing-impaired listeners. Five listeners with sensorineural hearing loss heard sentences spoken by three talkers in either a conversational style (“in the same manner in which [the talker] spoke in ordinary conversation,” p. 97) or a clear speaking style (“as clearly as possible, as if [the talker was] trying to communicate in a noisy environment or with an impaired listener,” p. 97). Sentence intelligibility was substantially higher for materials spoken clearly. Subsequent acoustic analyses of these materials revealed that clear speech was generally spoken slower, louder, and more clearly articulated compared to conversational speech, among other differences (Picheny et al., 1986). Since these foundational reports, clear speech has been reported to aid speech intelligibly for wide ranges of populations and listening conditions (e.g., Payton et al., 1994; Uchanski et al., 1996; Bradlow and Bent, 2002; Krause and Braida, 2002) [see Uchanski (2005) and Smiljaniç (2021) for reviews].

While clear speech comprises a constellation of acoustic modifications relative to conversational speech, the dimension of interest here is its slowed speaking rate. This decrease can be substantial (Picheny et al., 1986; but see Krause and Braida, 2002, 2004) and contributes to improved speech intelligibility (Uchanski et al., 1996; Ferguson and Kewley-Port, 2007). Changes in speaking rate can alter perception of speech beyond its intelligibility. Recognition of a given speech sound or word can be influenced by the speaking rate or duration of sounds surrounding it. These are known as temporal contrast effects (TCEs; also termed speaking rate normalization). For example, a target word might perceptually vary between “deer” and “tier” depending on the voice onset time (VOT) of its initial consonant (with “deer” perceived for shorter VOTs and “tier” for longer VOTs). When neighboring speech sounds are spoken at a slower rate, the target word is more likely to be perceived as having a shorter VOT; when neighboring speech sounds are spoken at a faster rate, the target word is more likely to be perceived as having a longer VOT (e.g., Miller and Liberman, 1979; Summerfield, 1981). Temporal context influences perception of a variety of speech sound contrasts in English and other languages [see Stilp (2020b) for review].

A common approach to investigating TCEs is to synthetically change the speaking rate of the speech materials. For instance, a particular sentence might be manipulated to decrease its speaking rate (slow rate condition) or increase it (fast rate condition). This way, all other things are held equal except for speaking rate (and, consequently, duration). This provides high experimental control while potentially losing some of the naturalness of the stimulus materials. Calls have been issued that the best way to understand how sensory systems operate is to present naturalistic stimuli (or stimuli that are as naturalistic as possible; e.g., Einhauser and König, 2010; Felsen and Dan, 2005). Using more naturalistic stimuli undoubtedly adds variability to stimulus materials, but this variability may be desirable since it more closely reflects the signals that are encountered in everyday perception. Recent studies of acoustic context effects in speech perception have used more ecological approaches (Stilp and Assgari, 2019, 2021; Reinisch, 2016) but have not considered the natural speaking rate variation commonly observed in clear versus conversational speech.

The present experiment sits at the intersection of these areas, measuring whether naturalistic clear and conversational speech elicited TCEs in speech perception. In one condition, listeners heard a context sentence that was spoken in either a clear or a conversational manner and then identified the following target word as “deer” or “tier.” We hypothesized that the different speaking rates across clear and conversational speaking styles would produce TCEs in word recognition. In a second condition, context sentence stimuli were constructed synthetically by digitally slowing the conversational sentence until it matched the duration and rate of the clear sentence. Here, all stimulus properties were held equal except for speaking rate (and, concomitantly, duration). We hypothesized that acoustic context effects using natural and synthetically slowed renditions of the same sentence would be larger than those produced by naturally produced clear and conversational sentences. This is consistent with the results of Stilp and Assgari (2019, 2021) in which tightly controlled stimuli show larger contrast effects than naturalistic, and therefore more acoustically variable, sentences.

## 2. Methods

### 2.1 Participants

Twenty-three undergraduate students participated in exchange for course credit. All listeners self-reported being native English speakers with no known hearing impairments.

### 2.2 Stimuli

#### 2.2.1 Context sentences

Picheny et al. (1986) noted that the decreased speaking rate of clear speech could be achieved through slower enunciations and/or increased use of pauses while speaking. Here, stimulus selection prioritized speaking rates that were altered via slower enunciations rather than insertion of pauses. Stimuli were selected from the Ferguson (2004) Clear Speech Database. A male talker (talker code M06) saying “Jean bought a bead at the store” was selected. The conversational version of this sentence had a duration of 1.34 s (speaking rate=5.22 syllables/s). The clear version of this sentence had a duration of 3.35 s (rate=2.09 syllables/s). These were used as the context sentences in the clear/conversational sentence block. To facilitate comparisons of natural versus synthetic changes in speaking rates, the conversational sentence was slowed by multiplying its duration by 2.5 in PRAAT (Boersma and Weenink, 2021) to make its final duration (and, thus, speaking rate) match that of the clear sentence. These renditions of the conversational sentence were used as context stimuli in the slowed conversational/conversational sentence block.^1^

Clear and conversational sentences can also vary in their intensities. Given the modest difference in intensities between the clear and conversational sentences chosen (1.63 dB), and that it was in the opposite direction of the trend identified by Picheny et al. (1986) (i.e., higher intensities in clear speech), root mean square intensities of all context sentences were equated.

#### 2.2.2 Target words

The last author was recorded saying “deer.” Using the synthesis methods of Winn (2020), a ten-step series of target words perceptually varying from “deer” to “tier” was created in PRAAT (Boersma and Weenink, 2021).^1^ This synthesis script progressively increased VOT from 21 ms in the “deer” endpoint to 82 ms in the “tier” endpoint. Secondary acoustic variations included total duration (lengthening from 488 to 512 ms across the continuum from “deer” endpoint to “tier” endpoint) and overall intensity (a decrease of 1.4 dB across the continuum as the initial stop consonant transitioned from being voiced to voiceless).

### 2.3 Procedure

The experiment was conducted online using Gorilla experiment builder (Anwyl-Irvine et al., 2020). After providing informed consent, listeners completed a six-trial headphone screen (Woods et al., 2017), reporting which of three tones on each trial was the quietest. The correct answer is the tone presented −6 dB relative to the other tones; the foil tone is presented 180° out-of-phase across channels and heard as quieter over loudspeakers due to destructive interference. Three of the 23 listeners failed the headphone screen twice. The headphone check was utilized as a means of standardizing stimulus presentation in an online experiment, and none of the sounds in the main experiment were dependent on headphone presentation. Therefore, these three listeners’ data were included in analyses. All statistical analyses reported below maintain whether these listeners are included or excluded from analyses.

Next, listeners completed 20 practice trials. On each trial, the context sentence “This time, I want you to click” spoken by the last author at a moderate rate (3.47 syllables/s) preceded an endpoint of the “deer”–“tier” target continuum. Feedback was provided after each trial. A performance criterion of ≥80% correct was required to continue to test; if this criterion was not met, the listener completed the practice block up to two more times. One listener failed to meet this criterion in any of their three attempts; their data were removed from all subsequent analyses.

Participants then proceeded to the main experiment, which consisted of two blocks: clear/conversational context sentences or slowed conversational/conversational context sentences. Each block included 160 trials (2 context sentences × 10 target words × 8 repetitions). Blocks were tested in a counterbalanced order across listeners, and trials within each block were presented in a different random order for each listener.

## 3. Results

An inclusion criterion of 80% correct on continuum endpoints was implemented for a given block to be included in statistical analyses. All listeners met this criterion in both blocks. Trial-level responses were analyzed using a generalized linear mixed-effects model using the lme4 package (Bates et al., 2014) in R (R Development Core Team, 2021).^1^ The outcome variable was binary (“deer” response=0, “tier” response=1). Fixed effects in this model included target word (mean-centered), speaking rate [sum-coded, conversational sentence coded as −0.5 and the comparatively slower context sentence (clear or slowed conversational) coded as +0.5], block (sum-coded; clear/conversational block coded as −0.5 and slowed conversational/conversational block coded as +0.5), and all interactions. The model building process began with these fixed effects and random intercepts for listeners. Random effects were added one at a time and tested via χ^2^ goodness-of-fit tests. If the added term explained significantly more variance, it was retained. The final random effects structure included random slopes for target and block with random intercepts for listeners. All modeling was conducted using the bobyqa optimizer to aid convergence.

**Fig. 1.**
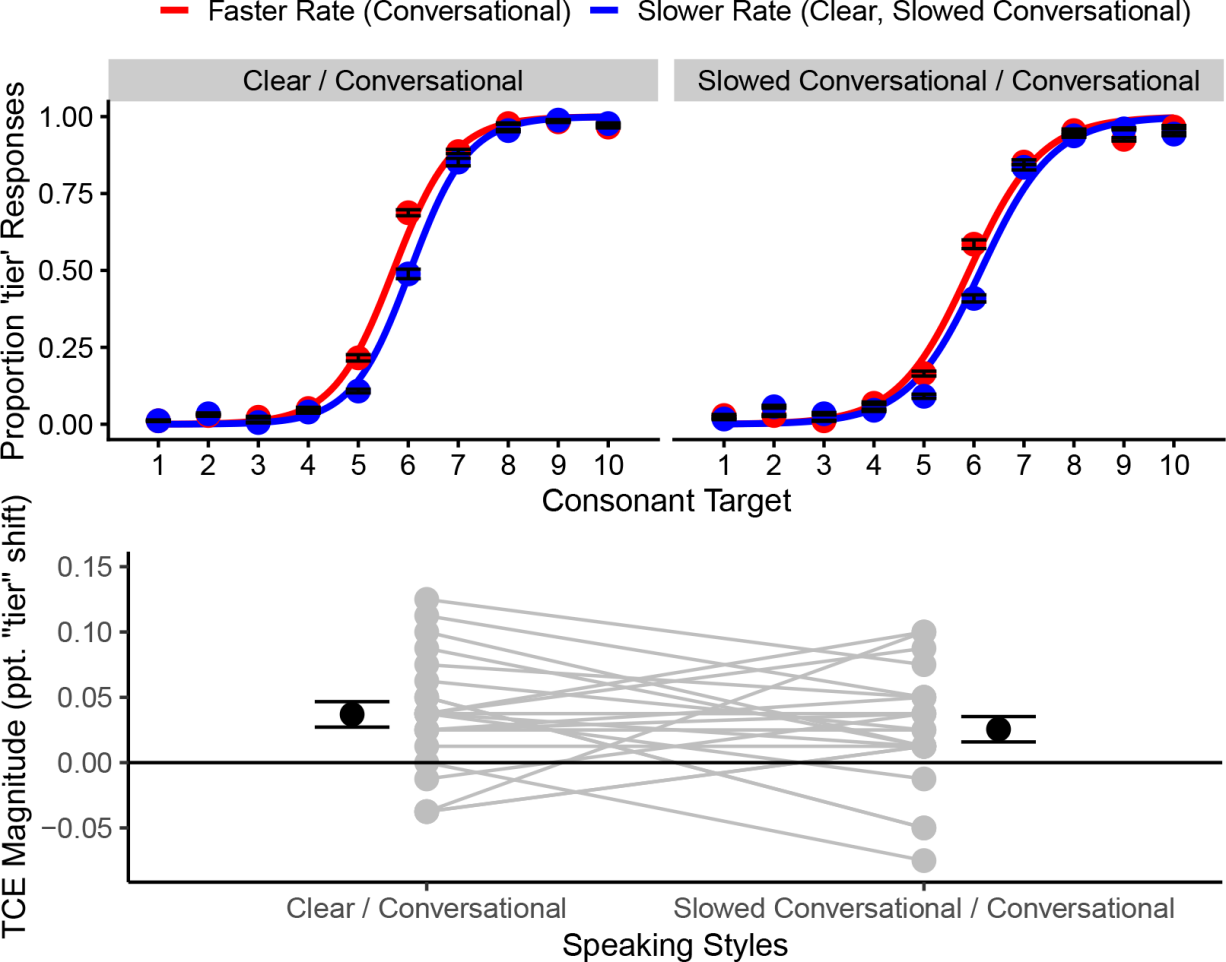
Top row: Mean proportions of “tier” responses to each target stimulus, varying from “deer” endpoint (1) to “tier” endpoint (10). Error bars depict ±1 standard error (SE). Mean responses in the clear/conversational block are depicted on the left; mean responses in the slowed conversational/conversational block are depicted on the right. Bottom row: TCEs calculated for each participant as the proportion shift in “tier” responses following the conversational context sentence compared to following the slower-rate (clear, slowed conversational) context sentence. Each gray circle depicts an individual participant. Black circles and error bars depict block means ±1 SE.

First, two models were run with block coded categorically (once with each level coded as the default) to test the fixed effect of rate against 0, thereby establishing the presence of a TCE. Statistically significant TCEs occurred in both the clear/conversational block $\left(\overset{ˆ}{\beta }=-0.60,\;Z=-4.34,\;p<0.0001\right)$ and the slowed conversational/conversational block $\left(\overset{ˆ}{\beta }=-0.31,\;Z=-2.49,\;p=0.013\right)$. Block was then coded via sum coding as described above. The model fit to behavioral data is illustrated in Fig. 1. Listeners responded “deer” more often overall (54.49% of responses; intercept: $\overset{ˆ}{\beta }=-0.68,\;Z=-4.27,\;p<0.0001$) but increased their “tier” responses as VOT in the target words increased (target: $\overset{ˆ}{\beta }=1.54,\;Z=13.74,\;p<0.0001$). Listeners responded “tier” less when the speaking rate was changed from faster (conversational) to slower (clear, slowed conversational), consistent with the predicted direction of TCEs (rate: $\overset{ˆ}{\beta }=-0.45,\;Z=-4.89,\;p<0.0001$). Contrary to predictions, TCE magnitudes did not differ across the two blocks (rate × block interaction: $\overset{ˆ}{\beta }=0.29,\;Z=1.57,\;p=0.12$). This is illustrated in Fig. 1, where individual listeners’ TCEs [calculated as the shift in percent “tier” responses following the conversational context sentence minus those following the slower (clear, slowed conversational) sentence] and the block means are shown (clear/conversational TCEs: mean=3.69% shift, SE=0.97; slowed conversational/conversational TCEs: mean=2.56% shift, SE=0.97). The only other significant fixed effect was the interaction between target and block $\left(\overset{ˆ}{\beta }=-0.31,\;Z=-4.92,\;p<0.0001\right)$, indicating that the psychometric function slopes were shallower in the slowed conversational/conversational block than in the clear/conversational block. Supplementary analyses did not reveal any systematic changes in responses to the conversational sentence across blocks nor to the slower-duration (clear, slowed conversational) sentence across blocks.^1^

## 4. Discussion

When instructed to speak clearly as in challenging listening conditions or to a listener with hearing difficulties, one of the prevailing acoustic differences talkers deploy is to slow their speaking rate (clear speech) relative to how they speak in better listening conditions (conversational speech; Picheny et al., 1986). In separate research, variation in speaking rate can alter perception of key temporal characteristics of speech through TCEs (or speaking rate normalization). Here, we hypothesized that sentences in clear and conversational speaking styles would promote speaking rate normalization, altering perception of voice onset time in the target words “deer” and “tier.” Results were consistent with this hypothesis (Fig. 1, top left).

Clear and conversational speech can differ in many acoustic properties, not only speaking rate (Picheny et al., 1986). When listeners heard the clear and conversational renditions of the context sentences in that testing block, several acoustic properties were varying across these items. Acoustic variability across context sentences can diminish the magnitude of the resulting context effect (Stilp and Assgari, 2019, 2021). We hypothesized that the context effect would be smaller following sentences with more acoustic variability (clear and conversational sentences) than sentences with minimal variability (conversational and slowed conversational sentences). However, data did not support this hypothesis; context effect magnitudes did not differ across conditions (Fig. 1, top right). One reason why this might have happened is that Stilp and Assgari (2019, 2021) made their observation of smaller context effects following naturalistic (as opposed to highly controlled) sentences across many experiments, as opposed to the single result being considered here. Additionally, Stilp and Assgari (2019, 2021) measured spectral context effects and not TCEs. These effects are subserved by different neural mechanisms [spectral contrast effects: neural adaptation (e.g., Stilp, 2020a); TCEs: neural oscillatory entrainment (Bosker and Ghitza, 2018) or evoked responses to acoustic edges (Kojima et al., 2021)] and are not obligated to follow the same patterns of results. Future investigation will elucidate which of these (or other) causes explain the lack of support for the second hypothesis.

**Fig. 2.**
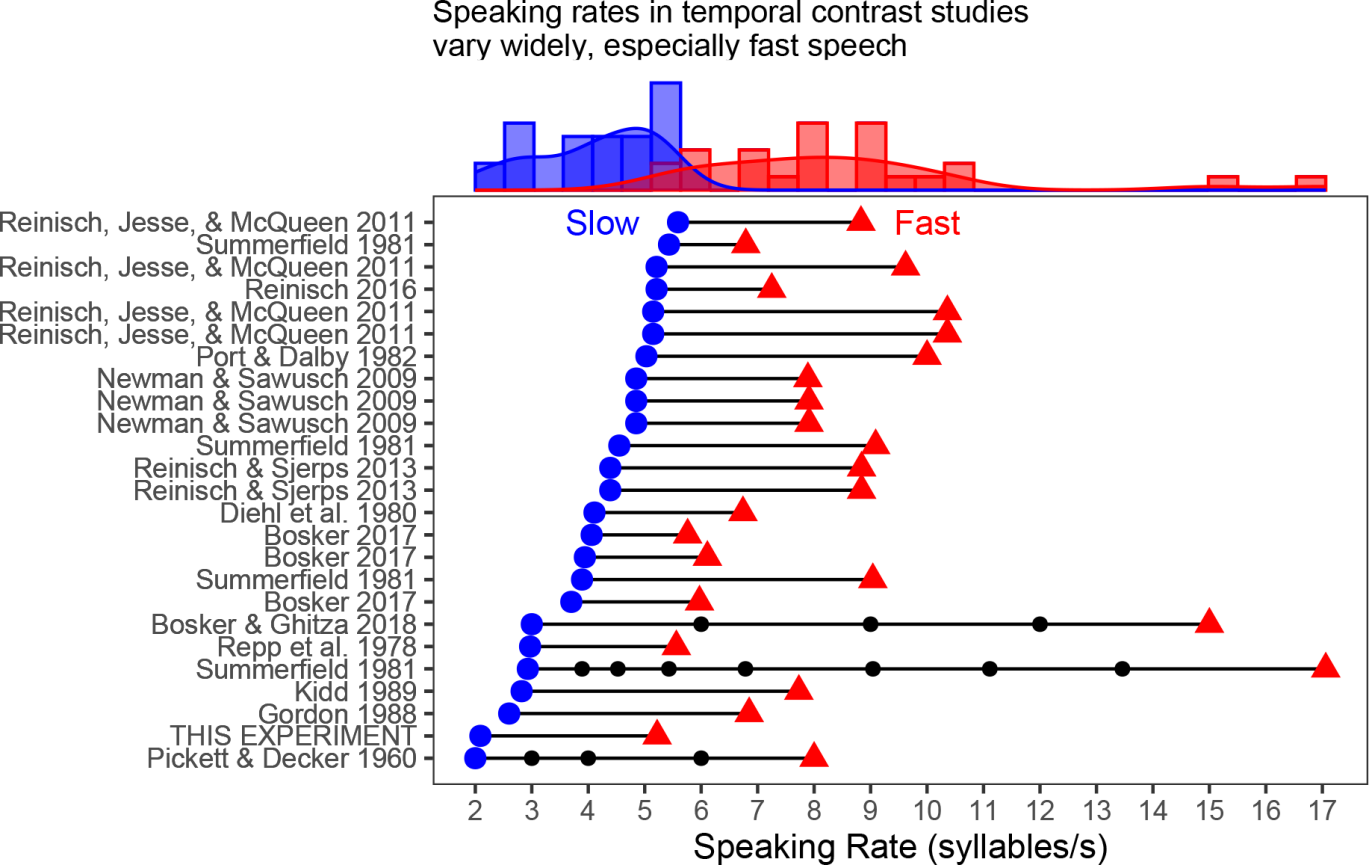
Slow (blue circles) and fast (red triangles) speaking rates tested in 25 experiments across 14 published reports (including this one) examining TCEs in speech perception. Rows are organized from bottom to top in order of increasing speaking rate (calculated as number of syllables divided by total duration) for slow sentences. Marginal histograms at the top capture the distributions of slow and fast speaking rates tested. Black circles indicate studies that tested more than two speaking rates in a given experiment; only the slowest and fastest rates tested were included in the histograms.

The magnitudes of TCEs in the present experiment were relatively small. This appears to be due to the narrow range of speaking rates employed (conversational sentence: 5.22 syllables/s, clear sentence: 2.09 syllables/s). Figure 2 illustrates rates tested in several other published reports of TCEs in speech perception [all of which were cited in Stilp’s (2020b) review] for which sufficient detail was provided to allow calculation of speaking rates (Pickett and Decker, 1960; Repp et al., 1978; Diehl et al., 1980; Summerfield, 1981; Port and Dalby, 1982; Gordon, 1988; Kidd, 1989; Newman and Sawusch, 2009; Reinisch et al., 2011; Reinisch and Sjerps, 2013; Reinisch, 2016; Bosker, 2017; Bosker and Ghitza, 2018). These speaking rates (mean slow speaking rate=4.11 syllables/s, mean fast rate=8.51 syllables/s) differ by larger amounts and extend to faster overall speaking rates than those tested here. The magnitude of the perceptual shift produced by the TCE increases monotonically as a function of speaking rate (Pickett and Decker, 1960; Summerfield, 1981), so it is possible that larger differences between the slow and fast speaking rates than those tested here would produce larger TCEs. Talkers in the Ferguson (2004) corpus were instructed for their speech to be “as much like your normal conversational style as possible” or to “speak clearly so that a hearing-impaired person would be able to understand you” (p. 2336). Such broad instructions were deliberate as to not elicit speech being spoken at faster rates than the talkers would use in ordinary conversation (and instructions can vary acoustic properties of clear speech; Lam et al., 2012; Lam and Tjaden, 2013). As a result, the difference between speaking rates for clear and conversational speech is not as large as those tested in slow and fast speech in other studies, which consequently produced a smaller TCE magnitude than those previously reported.

**Fig. 3.**
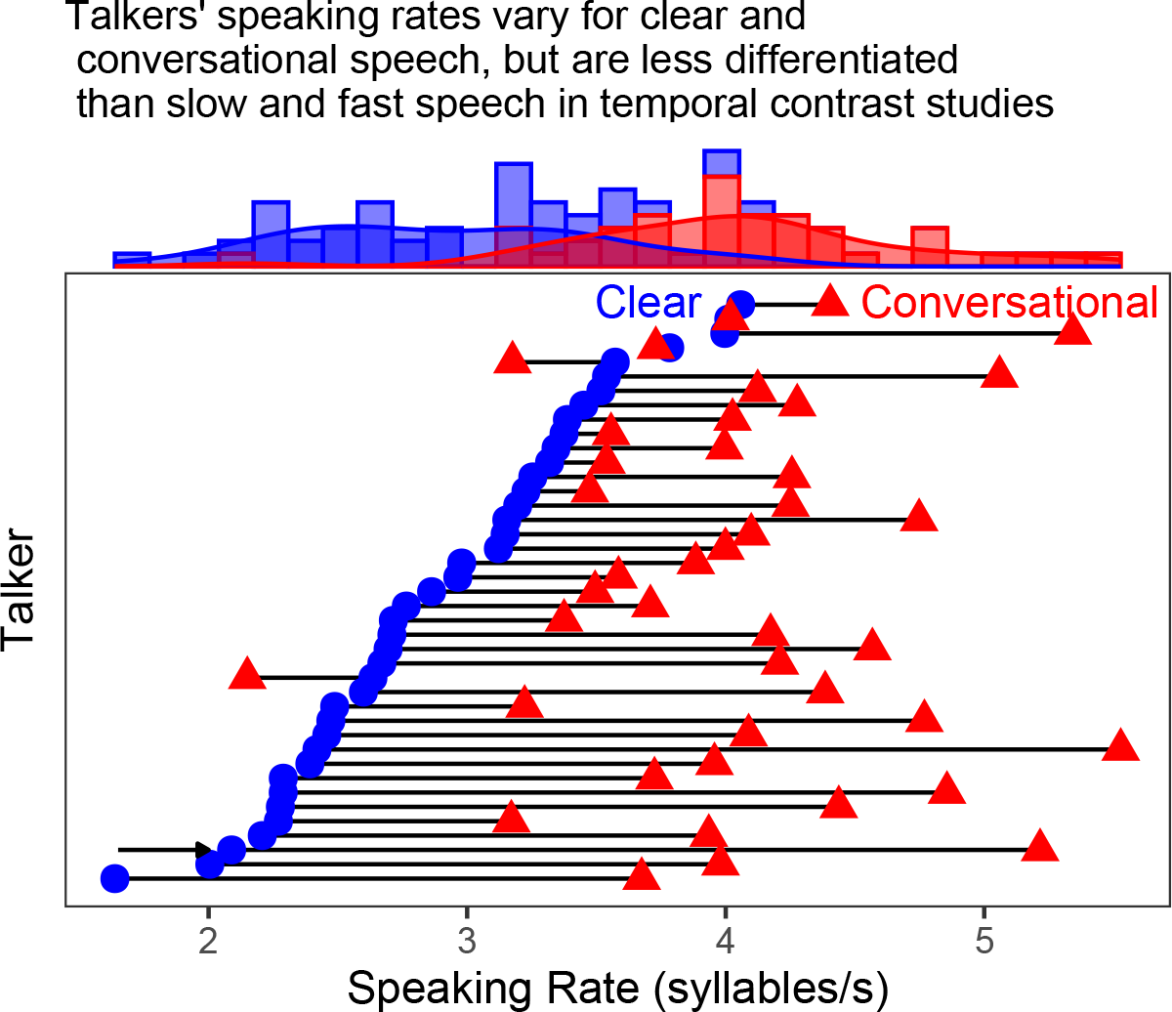
Speaking rates for clear (blue circles) and conversational (red triangles) renditions of the sentence “Jean bought a bead at the store” in the Ferguson (2004) database. Each row depicts clear and conversational speaking rates (calculated as number of syllables divided by total duration) for a single talker. The arrow in the third-to-bottom row depicts the talker and speaking rates for the materials tested in the present experiment. Rows are organized from bottom to top in order of increasing speaking rate for clear sentences. Marginal histograms at the top capture the distributions of speaking rates for clear and conversational renditions of this sentence. Note the significantly narrower range of values along the *x* axis compared to Fig. 2.

This research encourages reflection on two broader issues. First, while positive results of clear and conversational speech producing TCEs are reported here, how often is this happening in everyday listening? The clear and conversational sentence tokens tested here were selected owing to the variation in speaking rate through slower enunciation and not the elongation of pauses; these speaking rates are depicted in the third-to-bottom row of Fig. 3. Remaining rows of Fig. 3 illustrate other talkers’ speaking rates for clear and conversational versions of the same sentence in the Ferguson (2004) database. Tremendous variability exists across talkers’ speaking rates for clear speech and for conversational speech, and not every talker varies speaking rate to the same degree. The talker tested in this experiment exhibited the largest change in speaking rate across clear and conversational sentences in this database (as captured by the ratio of conversational speech rate to clear speech rate). Other talkers changed speaking rate by similar or smaller amounts, some not at all, and a few others in the opposite direction. Again, instructions for recording these materials did not explicitly ask talkers to slow or speed their speech (Ferguson, 2004), so this variability is perhaps not surprising. This variability might guide expectations of how often clear and conversational speech produce TCEs in everyday speech perception. Changes in speaking style might not elicit these context effects in every situation but could depend more so on how much the speaker alters speech rate (if at all).

The other issue of interest is the downstream consequences of clear and conversational speech for producing TCEs. A primary objective of clear speech is to make the talker more intelligible for the listener. Slowing speaking rate reduces the listener’s expended effort to understand the talker (Winn and Teece, 2021). Analogously, context effects might also decrease expended listening effort because they disambiguate phonemically ambiguous speech productions. However, these factors might not always align and could, in fact, compete with each other. Slowing speaking rate may aid intelligibility while also promoting perception of temporal characteristics of speech as being relatively faster, such as shorter VOT in the present experiment. As a result, the talker could be promoting recognition of words other than what they intended. If the talker is speaking slowly (clearly) and saying “tier,” the resulting TCE may be promoting perception of the word “deer” instead. These examples of “deer” and “tier” are acoustically similar but very semantically different, and listeners excel at using semantic context to aid speech understanding (e.g., Bilger et al., 1984; Pichora-Fuller et al., 1995; Winn and Teece, 2021). Other examples with both high acoustic and semantic similarity will provide stronger challenges to speech understanding. An important direction for future research is to explore when clear speech and temporal context effects, both of which intend to aid speech intelligibility, unintentionally come into conflict with one another and introduce a new challenge for understanding speech.

## Footnotes

1 All stimuli, data, analysis scripts, and supplementary analyses are available at https://osf.io/uq29c.
